# Snakes on a plain: biotic and abiotic factors determine venom compositional variation in a wide-ranging generalist rattlesnake

**DOI:** 10.1186/s12915-023-01626-x

**Published:** 2023-06-06

**Authors:** Cara F. Smith, Zachary L. Nikolakis, Kathleen Ivey, Blair W. Perry, Drew R. Schield, Neil R. Balchan, Joshua Parker, Kirk C. Hansen, Anthony J. Saviola, Todd A. Castoe, Stephen P. Mackessy

**Affiliations:** 1 Department of Biological Sciences, University of Northern Colorado, 501 20th Street, Greeley, CO 80639 USA; 2 Department of Biochemistry and Molecular Genetics, University of Colorado Denver, 12801 East 17th Avenue, Aurora, CO 80045 USA; 3 Department of Biology, University of Texas at Arlington, 501 S. Nedderman Drive, Arlington, TX 76019 USA; 4 Current address: Department of Ecology & Evolutionary Biology, University of Colorado, 1900 Pleasant Street, Boulder, CO 80309 USA; 5 Fresno City College, 1101 E. University Avenue, Fresno, CA 93741 USA

**Keywords:** Adaptive trait, Diet, Phenotypic variation, Proteomics, Toxins

## Abstract

**Background:**

Snake venoms are trophic adaptations that represent an ideal model to examine the evolutionary factors that shape polymorphic traits under strong natural selection. Venom compositional variation is substantial within and among venomous snake species. However, the forces shaping this phenotypic complexity, as well as the potential integrated roles of biotic and abiotic factors, have received little attention. Here, we investigate geographic variation in venom composition in a wide-ranging rattlesnake (*Crotalus viridis viridis*) and contextualize this variation by investigating dietary, phylogenetic, and environmental variables that covary with venom.

**Results:**

Using shotgun proteomics, venom biochemical profiling, and lethality assays, we identify 2 distinct divergent phenotypes that characterize major axes of venom variation in this species: a myotoxin-rich phenotype and a snake venom metalloprotease (SVMP)-rich phenotype. We find that dietary availability and temperature-related abiotic factors are correlated with geographic trends in venom composition.

**Conclusions:**

Our findings highlight the potential for snake venoms to vary extensively within species, for this variation to be driven by biotic and abiotic factors, and for the importance of integrating biotic and abiotic variation for understanding complex trait evolution. Links between venom variation and variation in biotic and abiotic factors indicate that venom variation likely results from substantial geographic variation in selection regimes that determine the efficacy of venom phenotypes across populations and snake species. Our results highlight the cascading influence of abiotic factors on biotic factors that ultimately shape venom phenotype, providing evidence for a central role of local selection as a key driver of venom variation.

**Supplementary Information:**

The online version contains supplementary material available at 10.1186/s12915-023-01626-x.

## Background

Understanding the processes that generate and maintain phenotypic diversity is central to understanding how natural selection shapes the evolution of traits and the variation in traits observed across populations. Predator–prey dynamics are a core feature of ecological systems, and the traits involved in these antagonistic interactions are under strong selective pressures that may give rise to novel, extreme, and complex phenotypes, which may also vary substantially across populations [1, 2] The resulting trophic adaptations that mediate predator–prey relationships represent a coalescence of morphological, behavioral, and physiological characteristics whose adaptive qualities are context-dependent and respond to a variety of influences [3]. Elucidating the biotic and abiotic determinants that shape these traits requires an integrative approach that examines possible evolutionary influences, their relationship to one another, and their combined effects on the individual components that comprise complex phenotypes.

A variety of extreme trophic traits have evolved among snakes, including a chemical means of prey capture via the production, storage, and delivery of heterogeneous mixtures of toxins in venom-producing lineages. As such, snake venoms provided a rich system for the investigation of the evolutionary drivers and mechanisms shaping complex trophic adaptations due to their clear genotype–phenotype relationships and their potent measurable bioactive properties [4, 5]. Though evolutionary history tends to correspond with broad compositional trends in venom among venomous snake lineages [2, 6, 7], venoms may also vary substantially within species and populations [8–22]. What factors drive venom variation, particularly within species, remains poorly understood [23, 24].

The association between diet and venom variation in some species points to prey availability as a major biotic determinant of venom composition and variation [22–37]. Indeed, the multifaceted influence of diet has been demonstrated by the expression of prey-specific toxins [25–27, 35, 37], correlations between toxinological diversity and prey diversity [2, 28], and the co-evolutionary dynamics of some populations to venom resistance in prey [23, 29, 38]. However, other studies have shown evidence that diet did NOT correlate with patterns of venom variation, indicating that other forces may also play major roles in shaping venom composition in different contexts [20, 39]. These varying conclusions indicate that the interplay of various determinants shaping venom composition is likely multifactorial, but only recently have venom studies explored the associations between biotic and abiotic factors and their relationship to venom phenotypes [19, 20]. Ultimately, the influences of population structure, gene flow, diet, and environment are complex and likely interacting, and it has remained unclear how these forces may synergistically contribute to shaping venom variation.

**Fig. 1:**
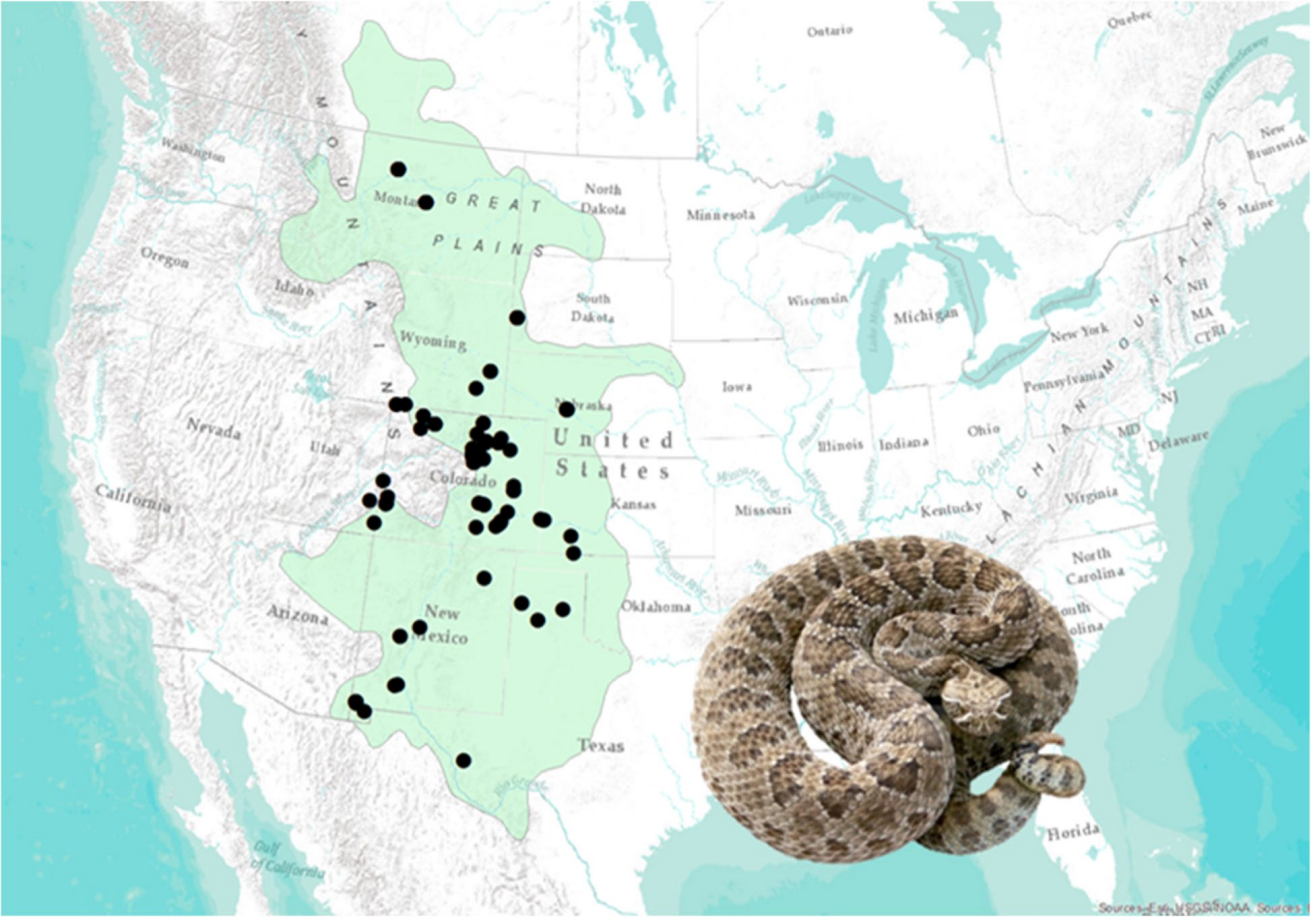
Known North American range of *C. v. viridis* (green) and sampling localities (black dots) used in this study (*n* = 146)

Here, we examine venom compositional diversity in a common, wide-ranging species and explore multiple potential drivers of variation in venom as observed across its range. The Prairie Rattlesnake (*Crotalus viridis viridis*) is a habitat generalist species found from northern Mexico, through the Great Plains of the western United States, to southern Canada (Fig. 1; [40]. Its venom is known to induce hemorrhage and tissue degradation due to the abundance of larger enzymes such as snake venom metalloproteases (SVMPs) and snake venom serine proteases (SVSPs), and smaller nonenzymatic myotoxin peptides result in tetanic paralysis of prey as well as myonecrosis [41–43]. Venom of the Prairie Rattlesnake has also been shown to contain many other classes of enzymes that are characteristic of rattlesnake venoms in general, including phospholipases A_2_ (PLA_2_), L-amino acid oxidases (L-AAO), and phosphodiesterases (PDE) [18], and ontogenetic shifts in venom composition, transitioning from a higher SVMP-level phenotype as juveniles to a myotoxin-rich venom as adults, occur in some populations [43]. Previous research on *C. v. viridis* venom has shown population-level differences in myotoxin presence [44] and PLA_2_ enzyme variation [45], but no studies have examined proteome-scale venom variation across the range of this species.

The current study uses a multipronged approach that examines phylogenetic and population genetic structure, diet, and environmental variables to provide a broad foundational context to understand factors that shape geographic patterns of venom variation. First, we characterize venom phenotypes range-wide with chromatographic profiling, shotgun proteomics, and biochemical and prey lethality assays. We then compare these patterns to range-wide phylogeographic structure, geographic trends in diet, and analyses of environmental niche variables and environmental niche modeling (ENM) to decipher the influences of biotic and abiotic factors that likely shape variation in venom composition.

## Results

### Venom collection

In total, 200 venom samples were collected from nine states (Fig. 1; Additional file 1: Table S1). Thirteen snakes collected were neonates (6.5%), 18 snakes were juveniles (9%), 21 were subadults (10.5%), and 147 were adults (73.5%). Because *C. v. viridis* venom composition varies ontogenetically [43], only adults were analyzed for trends in venom variation.

### High-performance liquid chromatography

**Fig. 2:**
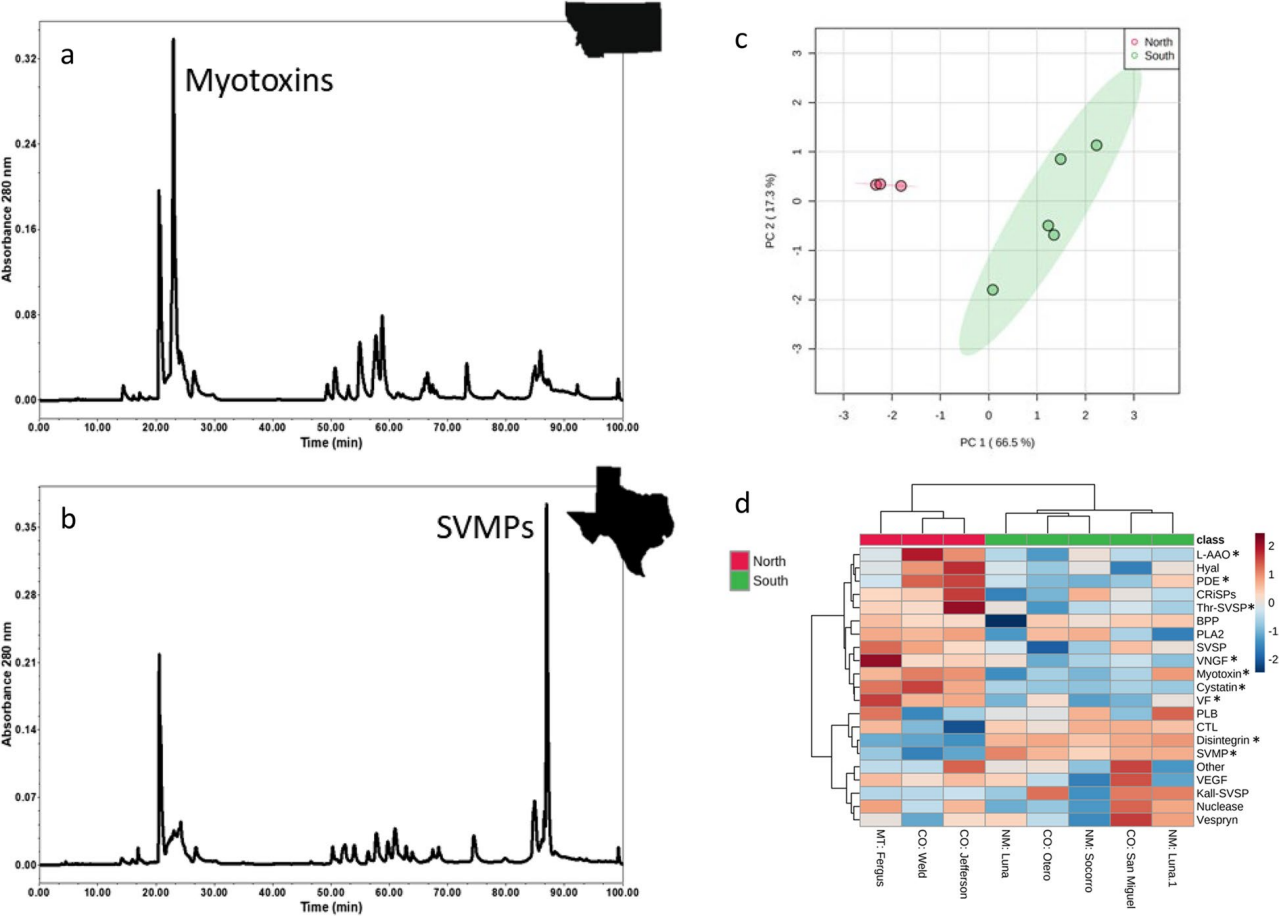
Reverse phase HPLC chromatograms showing a “northern” venom phenotype from Montana (**a**) and a “southern” venom phenotype from Texas (**b**). Note the size differences between the 22-min myotoxin peak and the 85-min SVMP peaks. **c** Principal component analysis with 95% confidence intervals of shotgun proteomics log-transformed protein spectral intensity data with proteins binned by protein family (*n* = 8). **d** Heatmap of log-transformed protein spectral intensities using Euclidean distance measures and Ward’s clustering method showing significantly different protein abundances (*) based on unpaired *T*-test (*p* < 0.05; *n* = 8). Red = northern phenotype, green = southern phenotype. L-AAO = L-amino acid oxidase, Hyal = hyaluronidase, PDE = phosphodiesterase, CRiSPs = cysteine rich secretory proteins, Thr SVSP = thrombin-like snake venom serine protease, BPP = bradykinin potentiating peptides, PLA2 = phospholipase A_2_, SVSP = snake venom serine protease, VNGF = venom nerve growth factor, VF = venom factor, PLB = phospholipase B, CTL = c-type lectin, SVMP = snake venom metalloprotease, VEGF = vascular endothelial growth factor, Kall-SVSP = kallikrein-like snake venom serine protease

HPLC analysis revealed complex toxin profiles from all snakes analyzed, and myotoxins eluted at approximately 22 min, SVSPs, cysteine-rich secretory proteins (CRiSPs), and PLA_2_s between 50 and 70 min, L-AAO at 78–79 min, and SVMPs between 85 and 90 min (Fig. 2a, b; based on Saviola et al., 2015 [43] and SDS-PAGE/MS data on peaks). Considerable complexity of toxin composition was observed in peaks that eluted between 50 and 70 min, so these percentages were not used in analysis because quantification of specific toxin families was unclear. The proportion of myotoxin ranged from 2.2 to 60.7% (average = 32.2, median = 35.3), L-AAO percentages ranged from 0 to 2.9% (average = 0.96, median = 0.9), and SVMP proportion ranged from 3.5 to 50.1% (average = 19.8, median = 13.4); for all of these peaks, the stated toxin family was exceedingly the most abundant representative. Snakes from northern regions (ex. Montana, Wyoming, Nebraska) displayed higher percentages of myotoxin and lower proportions of SVMPs (Fig. 2a) than snakes from southern areas (e.g., Texas, New Mexico; Fig. 2b). Snakes from southern regions in general had lower percentages of myotoxin and higher percentages of SVMPs (Fig. 2b). All percentages determined by area under the curve of HPLC chromatograms were used for geographic venom analysis using Mantel tests and linear regression analysis.

### Enzymology

The 147 adult venoms tested displayed high levels of variation in all enzymes. Adult SVMP specific activity ranged from 0.09 to 1.36 ΔA_342nm_/min/mg venom protein (average = 0.51, median = 0.39). Thrombin-like specific activity ranged from 346.1 to 2607.4 nmol product produced/min/mg venom (average = 1364.4, median = 1285.3) and kallikrein-like specific activity ranged from 64.6 to 2851.9 nmol product produced/min/mg venom (average = 1012.2, median = 962.1). Venoms ranged from 6.7 to 125.6 nmol product formed/min/mg venom (average = 42.7, median = 39.4) for L-amino acid oxidase activity. Phosphodiesterase activity ranged from 0.11 to 1.6 ΔA_400nm_/min/mg venom (average = 0.5, median = 0.46). Phospholipase A_2_ activity ranged from 4.7 to 72.3 nmol product/min/mg (average = 25.4, median = 22.3). These values were investigated for geographic patterns in activity with Mantel tests and linear regression analysis against latitude.

### Proteomics

Proteins identified from shotgun proteomic analyses were binned into toxin protein families, and total intensity for each family was compared across samples and used for principal component analysis. We detected 94 proteins across 20 protein families’ total (Additional file 2: Tables S2-S3). The toxin families with the largest number of proteins assigned to them were SVSPs [24], SVMPs [20], PLA_2_s [15], C-type lectins (CTL; 9), disintegrins [5], and myotoxins [4]. Venoms of snakes from New Mexico and southern Colorado had a higher abundance of SVMPs than those from northern Colorado and Montana, and the venom from the Montana snake had the highest amount of SVSPs. The individual from Socorro Co., NM, had the highest levels of PLA_2_, while the snakes from Luna Co., NM, had the lowest levels. CTLs were most abundant in NM snakes and least abundant in northern Colorado snakes. Disintegrin levels were highest in snakes from NM and southern Colorado. With the exception of one individual from Luna Co., snakes in New Mexico had the lowest levels of myotoxins. Myotoxin abundance was also lower in southern Colorado than northern Colorado or Montana. Principal component analysis revealed the presence of two distinct clusters that corresponded to a northern group containing MT and northern CO venoms and a southern group with southern CO and NM venoms (Fig. 2c). Between these groupings, we detected significantly higher abundances of cystatin (*p* < 0.001), L-AAO (*p* = 0.03), myotoxin (*p* = 0.03), PDE (*p* = 0.04), thrombin-like serine proteases (*p* = 0.04), VF (*p* = 0.01), and VNGF (*p* = 0.03) in northern snakes and higher levels of disintegrins (*p* < 0.001) and SVMP (*p* < 0.001) in southern snakes (Fig. 2d).

### Statistical analyses

Based on a Mantel test, the percentage of myotoxin and percentage of SVMPs significantly varied based on geographic location (*r* = 0.323, *p* = 0.001; *r* = 0.316, *p* = 0.001, respectively), while the percentage of L-AAO did not vary with geographic location (*r* = 0.032, *p* = 0.232). Of other enzymes tested, metalloproteases (*r* = 0.28, *p* = 0.001), thrombin-like serine proteases (*r* = 0.17, *p* = 0.001), and kallikrein-like serine proteases (*r* =  − 0.03, *p* = 0.016) showed significant geographic variation.

**Fig. 3:**
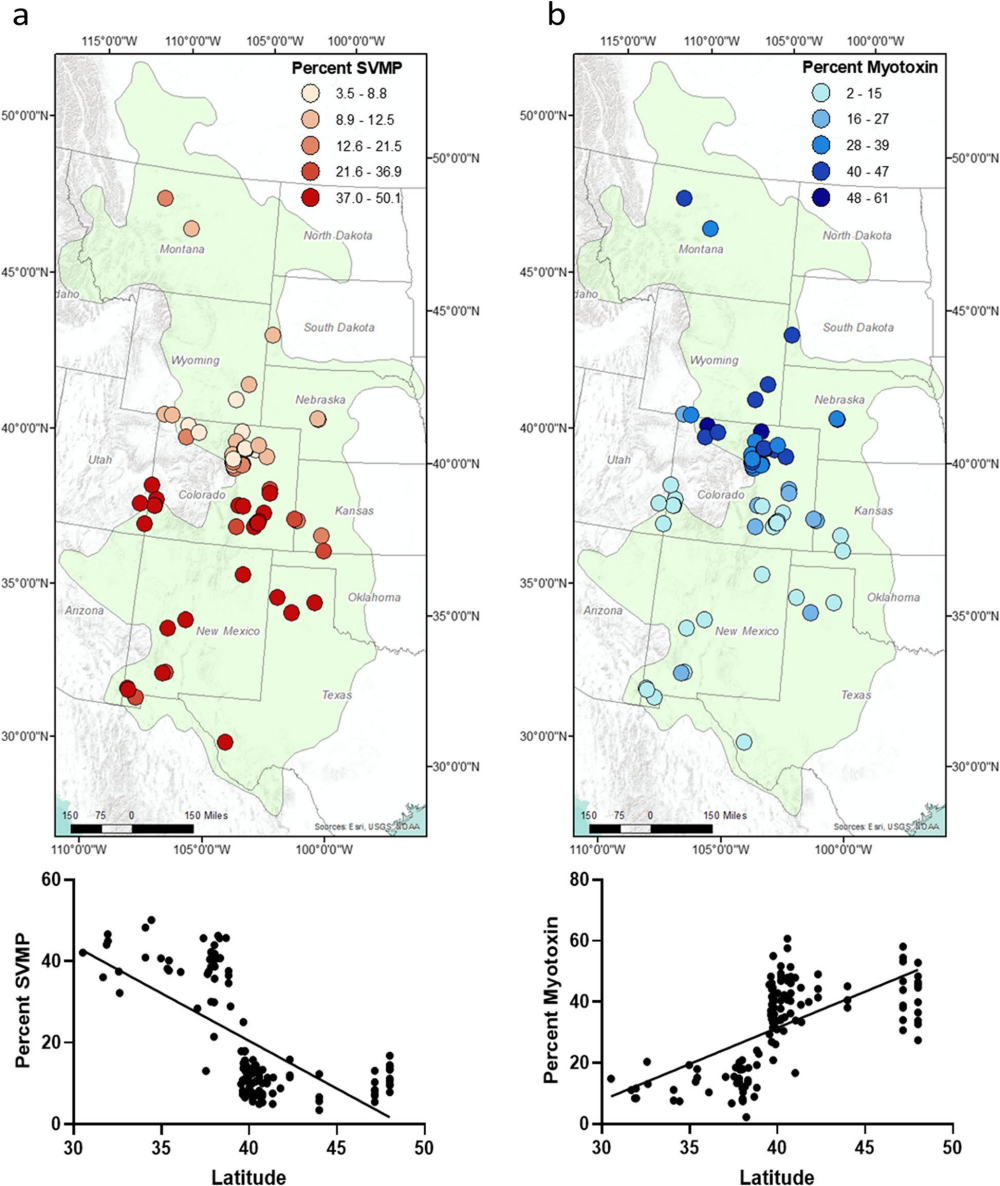
**a** Geographic distribution of percent SVMP and linear regression by latitude (*n* = 130). Darker red represents higher percentages of SVMP. **b** Geographic distribution of percent myotoxin and linear regression (*n* = 130). Darker blue represents higher percentages SVMP. Notice the dramatic shift in abundance for both toxins around central Colorado (~ 39th parallel north)

The percentage of SVMPs (Fig. 3a; *R*^2^ = 0.4412, *p* < 0.001) and the percentage of myotoxin (Fig. 3b; *R*^2^ = 0.408, *p* < 0.001) varied significantly with latitude in linear regression analysis. Strikingly, rather than an increasing or decreasing gradient of abundance, both toxins have an apparent high or low “plateau,” with a drastic shift in abundance occurring in central Colorado (approximately the 39th parallel north). Azocasein metalloprotease activity (Additional file 3: Figure S1a; *R*^2^ = 0.323, *p* < 0.001), thrombin-like (Additional file 3: Figure S1b; *R*^2^ = 0.181, *p* < 0.001), and kallikrein-like (Additional file 3: Figure S1c; *R*^2^ = 0.13, *p* < 0.001) serine protease activity, as well as phosphodiesterase activity (Additional file 3: Figure S1d; *R*^2^ = 0.052, *p* < 0.001), were significantly correlated with latitude. Neither PLA_2_ (Additional file 3: Figure S1e; *R*^2^ = 0.001, *p* = 0.65) nor L-AAO (Additional file 3: Figure S1f; *R*^2^ = 0.001, *p* = 0.68) activities significantly correlated with latitude. Using only toxins that were significant in the both the Mantel test and in the linear regressions, cluster analysis in PC-ORD 7.07 broadly defined two major venom phenotypes that explained 100% of the variation (Table 1). These groups represented a high-myotoxin venom profile and a high-SVMP (and also high SVSP) profile and were used in MAXENT analysis, which requires inputs to be categorical (by species typically, but in this case venom phenotype).

**Table 1:** Averages and standard deviations of significantly different toxin abundances and specific activities between northern (*n* = 108) and southern phenotypes (*n* = 38)

	Northern phenotype	Southern phenotype
Percent myotoxin	42 (7.2)	14 (5.3)
Percent SVMP	10.6 (3.3)	37.8 (7.8)
SVMP activity	0.34 (0.14)	0.98 (0.19)
Kall-SVSP activity	856 (563)	1481 (698)
Thr-SVSP activity	1262 (471)	1681 (464)

*Abbreviations*: *SVMP* snake venom metalloproteases, *Kall-SVSP* Kallikrein-like snake venom serine protease, *Thr-SVSP* thrombin-like snake venom serine protease. SVMP activity is expressed as ΔA_342nm_/min/mg venom protein, and Kall-SVSP and Thr-SVSP activities are expressed as nanomoles product produced/min/mg venom

### Phylogeographic and population genetic analyses

**Fig. 4:**
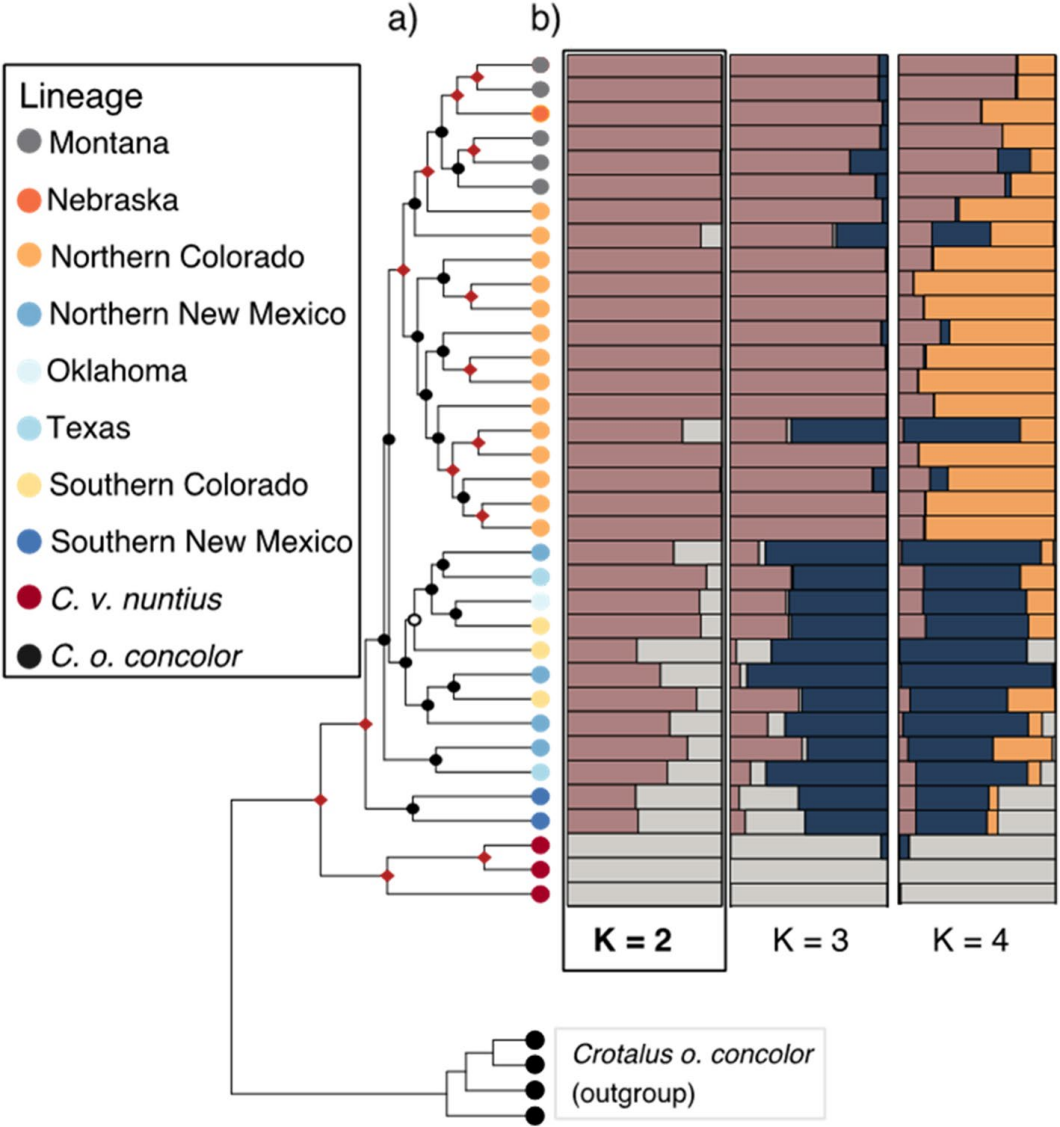
**a** Bayesian phylogeny generated from all nuclear SNPs in BEAST (*n* = 35). Nodes with > 0.95 posterior support are denoted with red diamonds. For nodes with less support, nodes are labeled with closed circles if posterior support is above 0.50 and open circles for nodes with posterior support below 0.50. **b** Mosaic plots from STRUCTURE results using all 35 samples from the nuclear dataset run from *K* = 2–4, where *K* = 2 was provided greatest support. Colored bars indicate the proportion of SNPs associated with each identified cluster. Note that mosaic plots and the nuclear phylogeny are aligned by sample

The phylogeny generated from our nuclear SNP dataset shows strong posterior support (> 0.95 PP) for a split between *C. v. nuntius* and *C. v. viridis*, with the *C. v. viridis* clade containing three subclades of varying support. One of these subclades corresponds to samples from more northern localities, including individuals from Montana, Nebraska, and northern Colorado; this northern subclade is supported with low posterior support (> 0.50 PP; Fig. 4a). Also, within the *C. v. viridis* clade, populations from southern Colorado, New Mexico, Texas, and Oklahoma form clades that diverge more basally within the larger *C. v. viridis* clade (Fig. 4a). Our population cluster results from STRUCTURE indicated that a *K* value of 2 was the best supported model based on the Evanno method (Δ*K*) across all *K* values tested (Fig. 4b), although we show results for additional values of *K* further to investigate evidence for potential structure across these populations. Results from the *K* = 2 model shows substantial differentiation of ancestry coefficients between *C. v. viridis* and *C. v. nuntius*, with moderate levels of admixture/differentiation between *C. v. nuntius* and southern *C. v. viridis* localities. Our results also show sub-structuring between northern and southern *C. v. viridis* samples for various models of *K* (*K* = 2–4), although no values of *K* tested clearly demarcate southern and northern populations of *C. v. viridis*. However, these analyses do highlight gradual shifts in population differentiation along a North–South axis of *C. v. viridis* populations. Thus, while STRUCTURE analyses provide some evidence for the cohesiveness of northern populations, they do not support a clearly defined break in population differentiation between northern and southern *C. v. viridis*.

### Diet

In total, 638 *C. v. viridis* specimens were examined from seven natural history collections. Stomach contents were recovered from 130 snake stomachs (20.4% of snakes) and comprised four broad taxonomic classes: Aves, Mammalia, Amphibia, and Reptilia. Of these prey classes, Mammalia was the best represented, comprising 78.2% of adult rattlesnake stomach contents. Lizards represented only 10.3% of all prey items recovered from adults; avian prey items were also recovered from five food boli. A single amphibian was found in an adult snake from Hidalgo Co., New Mexico. Other contents found (3.4% of total) included nematode roundworms as well as unidentifiable material likely not prey related.

**Fig. 5:**
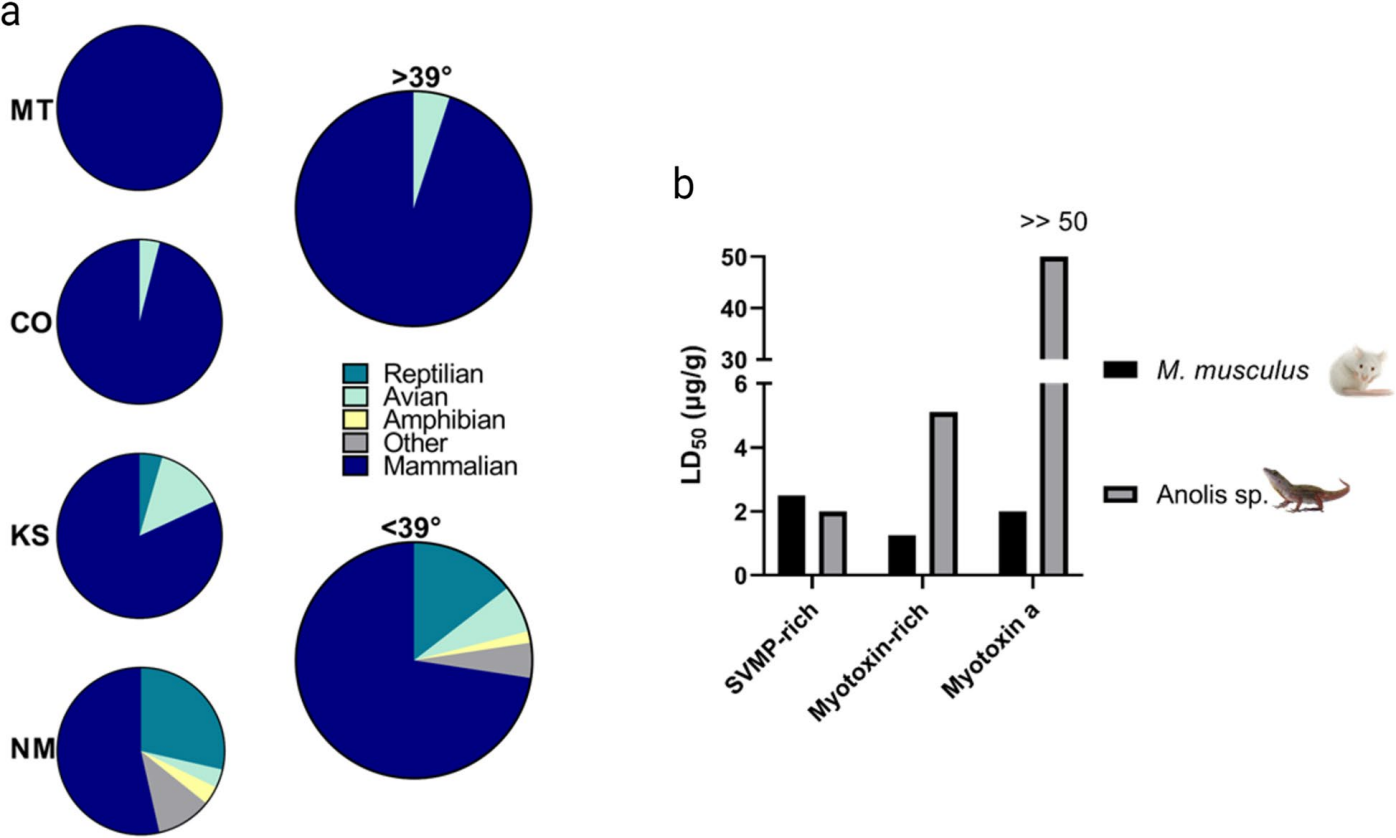
Diet of Prairie Rattlesnakes across the North American range and relation to venom phenotype. **a** Proportion of each prey class by state or grouped above (*n* = 20) or below (*n* = 64) the 39th latitude. Only states with more than one prey item are displayed. MT = Montana (*n* = 3), CO = Colorado (*n* = 25), KS = Kansas (*n* = 22), NM = New Mexico (*n* = 25). **b** Comparisons of LD_50_ values between SVMP-rich venoms, myotoxin-rich venoms, and purified myotoxin a against NSA mice and *Anolis* lizards

Only three prey items (all mammals) were recovered from Montana snakes (Fig. 5a). One mammal each was collected from Oklahoma, South Dakota, and Wyoming. Of the 25 prey items collected from Colorado, one was avian (4%) and 24 (96%) were mammalian. Three birds, one lizard, and 18 rodents were recovered from 22 snakes in Kansas (14%, 4.5%, 82%, respectively). Of the identifiable material from New Mexico snakes, one amphibian (4%), one bird (4%), 8 lizards (32%), and 15 (60%) mammals were recovered from 25 snakes (Fig. 5a).

Based on the northern and southern venom classifications that are separated by the 39th parallel north, we investigated the dietary composition of snakes above and below this latitude. The majority of prey items (95%) from snakes in the northern region were mammals, with only 1 bird recovered (Fig. 5a). The diet of snakes south of the 39th latitude was also dominated by mammals (72.6%); however, we also recovered reptilian (14.5%), amphibian (1.6%), and avian prey (6.5%), along with 4.8% unidentifiable material. Discriminant analysis based on latitude confirmed the dietary clusters identified as north and south of the 39th latitude that corresponded to north and south venom phenotypes (*p* < 0.001; *F* = 67.94).

We also found that diet has a significant relationship with venom toxin activities and composition overall (*F* = 207.9, *p* < 0.001), and diet showed a statistically significant relationship with the toxins that varied geographically (SVMP activity (*F* = 333), thrombin-like SVSP activity (*F* = 17.6) and kallikrein-like SVSP activity (*F* = 37), and percentage of myotoxin (*F* = 419) and percentage of SVMP (*F* = 872); *p* < 0.001 for all).

### Lethality (LD_50_) assays

SVMP-rich venoms had LD_50_ values of 2.5 μg/g and 2.0 μg/g towards NSA mice and *Anolis* lizards, respectively (*n* = 12; Fig. 5b). Myotoxin-rich venoms were more toxic towards lab mice (1.25 μg/g; *n* = 12) than towards lizards (LD_50_ = 5.1 μg/g; *n* = 12). Purified myotoxin a was quite toxic towards mice (2.0 μg/g; *n* = 9) and was essentially nontoxic to lizards (> > 50 μg/g; *n* = 4).

### Environmental niche modeling

**Fig. 6:**
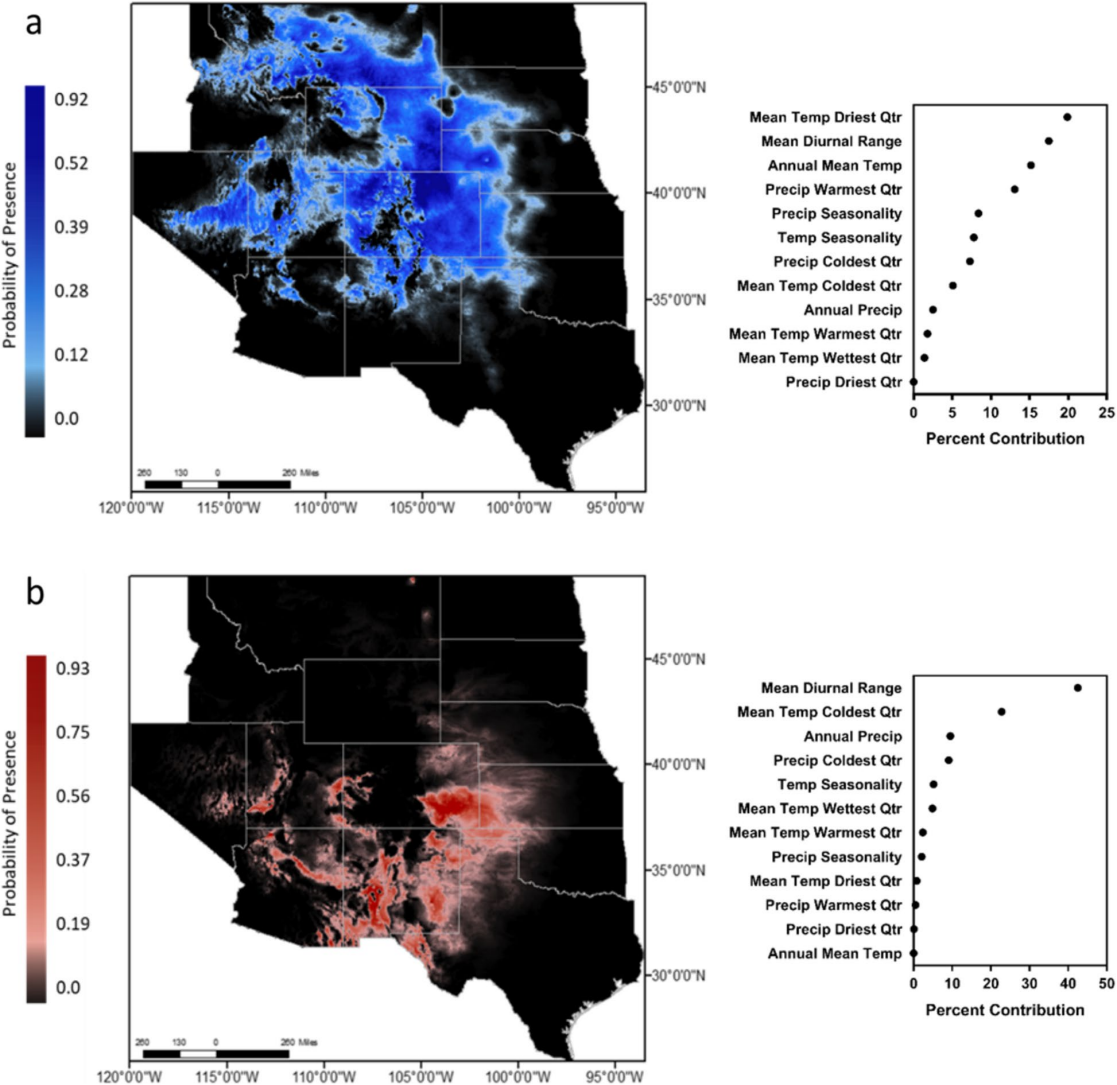
MAXENT calculated likelihood of occurrence and model response to the variables included for the **a** high myotoxin phenotype (*n* = 25) and **b** high SVMP phenotype (*n* = 28) in the western United States

The ENM area under the curve (AUC) was high for both the high myotoxin phenotype (AUC = 0.881) and the high SVMP phenotype (AUC = 0.942), indicating high predictive power of occurrence likelihood (Fig. 6a, b). The high myotoxin phenotype had a higher probability occurrence in the northern regions of the known range of *C. v. viridis* but extended towards northern New Mexico, northern Arizona, Utah, and eastern Nevada (Fig. 6a). The high SVMP phenotype had a higher probability of occurrence in the known southern range of this species (Fig. 6b) from Colorado to southern New Mexico and southwestern Texas. The predicted range extended partially into Utah and Nevada and had a high probability up to central Colorado.

The environmental variables responsible for these predicted distributions differed between venom phenotypes (Fig. 6a, b; Additional file 4: Table S4). Mean temperature of driest quarter (BIO 9) contributed the most to the range of the northern phenotype (percent contribution = 19.9%), followed by mean diurnal range (BIO 2; 17.5%) and annual mean temperature (BIO 1; 15.2%). Mean diurnal range contributed the most to the predicted southern phenotype distribution (BIO 2; 42.5%), followed by mean temperature of coolest quarter (BIO 11; 22.8%). Interestingly, the third most significant variable predicting the northern phenotype (BIO 1: annual mean temperature) contributed the least (0%) to predicting the southern phenotype.

Reptile and mammal richness was significantly predicted by environmental variables (*p* < 0.001; adjusted *R*^2^ = 0.947, *R*^2^ = 0.859, respectively). Specifically, reptile species richness was significantly correlated with BIO 1 (*p* < 0.001), BIO 2 (*p* = 0.001), BIO 4 (*p* < 0.001), BIO 10 (*p* < 0.001), BIO 11 (*p* < 0.001), BIO 15 (*p* < 0.001), BIO 17 (*p* < 0.001), BIO 18 (*p* = 0.044), and BIO 19 (*p* < 0.001), while mammal species richness was correlated only with BIO 12 (*p* < 0.001), BIO 17 (*p* = 0.008), and BIO 18 (*p* < 0.001). When determining relationships of dietary availability to venom phenotypes, there was a significant contribution to the final multinomial logistic regression model (*p* < 0.001) of both mammal species richness (*p* = 0.007) and reptile species richness (*p* < 0.001).

In order to investigate the influence of diet and environment on venom composition we performed hierarchical linear regressions with two predictor models: (1) only reptile and mammal species richness and (2) Species richness combined with environmental variables. Both models significantly predicted the percentage of myotoxin and SVMP (*p* < 0.001), but the second model had a higher *R*^2^ value in both cases (0.696 > 0.419, and 0.804 > 0.799, respectively). Azocasein SVMP, thrombin-like SVSP, kallikrein-like SVSP activities were also significantly predicted by both models (*p* < 0.001) with a higher *R*^2^ value for model 2 compared to model 1 (0.747 > 0.433; 0.273 > 0.1, 0.236 > 0.181, respectively). PDE activity and percentage and activity of L-AAO was only predicted by the second model (*p* < 0.001, *p* = 0.044, and *p* < 0.001, respectively) and PLA_2_ did not have a significant relationship with either model.

## Discussion

Unraveling the multifaceted and nuanced abiotic and biotic factors that shape complex phenotypes is a major hurdle to understanding complex trait evolution, and our results demonstrate the influence of two of these factors, diet and environment, in shaping wide-ranging geographic venom variation in the Prairie Rattlesnake. Previous systems have identified a relationship between temperature-related variables and venom composition; however, these patterns did not extend to diet [20]. Therefore, our results provide a critical link between abiotic and biotic influences and their combined effects on venom composition and demonstrate that selection on venom variation is context-dependent even in closely related populations.

We found two distinct venom phenotypes in *C. v. viridis*: a northern myotoxin-rich phenotype and a southern SVMP-rich phenotype, which are distributed along a latitudinal gradient that corresponds with climatic and prey variation. The diversity of components in pit viper venoms has been shown to correlate with dietary complexity [2]; however, this does not appear to be a trend in our study. In this case, both venom phenotypes are dominated by a single toxin family (~ 50% myotoxins or SVMPs), distantly followed by other toxins of much lower abundance. Our analyses also identified the toxin responsible (myotoxin a) for the mammal-specific toxicity observed in the northern phenotype, which to our knowledge is the first prey-specific toxin identified from a rattlesnake. These geographic patterns in venom variation in *C. viridis* add an intriguing new level of complexity to a broader trend observed in rattlesnake venom variation [18, 21, 46–48]. The dichotomy observed effectively represents a trade-off between rapid-acting neurotoxic venom (type 2) and enzyme-rich degradative venom (type 1; sensu [18]). Though the myotoxin-rich northern phenotype lacks the classical PLA_2_-based neurotoxin characteristic of type 2 venoms, its dominant component is a rapidly-acting toxin that causes near-immediate muscular tetanic paralysis [49].

The maintenance of type 1 and 2 venom phenotypes has been studied in other rattlesnake species and are thought to be driven by either disruptive selection against type 1 + 2 venoms [20] or localized directional selection for a particular venom type [19], resulting in only rare cases of intermediate phenotypes. Regardless of the underlying selection regime, we observe similar evidence in *C. v. viridis* for the presence of either type 1 or 2 venom across populations, and a rapid transition between these phenotypic extremes within a narrow geographic range of central Colorado, indicating a steep cline in adaptive advantage across this latitudinal threshold. Interestingly, we found some evidence for North–South variation in population genetic structure, although the present-day population genetic differentiation across this narrow region (central Colorado) is not strong or clearly defined. It is possible that past population structure across this North–South axis, which is now potentially obscured by more recent gene flow, may have contributed to the differentiation and divergence of venom phenotypes across this axis. It therefore remains an open question whether past population structure contributed to venom variation divergence, but evidence that population structure is not strong currently across this region suggests that this phenotype transition is likely maintained by relatively strong selection for divergent venom phenotypes on either side of this transition.

In spite of its large geographic range and substantial variation in venom composition, population genetic analyses suggest relatively little present-day population genomic structure across geographically distant populations of *C. v. viridis*. This absence of well-defined population structure yet high venom variation is similar to patterns observed in the closely related Mojave Rattlesnake (*C. s. scutulatus*; [19, 20]), which also possesses populations showing type 1 or type 2 venoms. Taken together, these similar examples suggest that selective forces that operate on venom variation are likely very strong, to the extent that distributions of these traits are not readily explained by population genetic structure, and instead that alleles that encode variants underlying venom composition may readily defy gene flow, due primarily to the impacts of local and regional selection regimes on related venom phenotypes.

Our findings highlight that a latitudinal shift in diet corresponds to a switch from SVMP-rich venom to myotoxin-rich venom. All gut contents from the northern range (Montana, South Dakota, northern Colorado) were rodents, with the exception of a single bird. A higher diversity of prey type and a considerably higher abundance of lizards were found in snakes from southern areas (New Mexico, southern Colorado, Kansas, Oklahoma). Prey availability and diversity play a critical role in the selective processes that shape venom composition in some systems [2, 31, 32]; therefore, it is important to note that these dietary patterns likely do not reflect a difference in *prey preference* but a difference in *prey availability*, as the proportion of available reptile prey species (particularly lizards) decreases precipitously moving north as a general rule [50–52]. In fact, only one species of lizard is found in the northern extreme of *C. v. viridis* range in southern Canada [53], making rodents the dominant food source available at higher latitudes (cf. 53). The relationship between diet and venom composition in *C. v. viridis* represents a departure from the trends displayed in the closely related *C. s. scutulatus*, where diet did not correlate with venom variation [20]; however, *C. s. scutulatus* does not reach the same northern latitudinal extremes of *C. v. viridis.*

*Crotalus s. scutulatus*, a sympatric rattlesnake in parts of the extreme southern range of *C. v. viridis*, still predominantly preys on mammals [20]; however, lizards have been shown to represent a much larger proportion of diet (up to 44% [54]). Another potentially sympatric snake in southern regions, *C. lepidus klauberi*, is found in more isolated and higher elevation regions and predominantly feed on lizards (55.4%), with a much lower proportion of diet consisting of mammals (13.8%; [55]). In a larger rattlesnake, *C. atrox*, sympatric in the southern range, mammals comprise the vast majority of diet (94.8%); however, this species is large enough regularly to consume adult lagomorph prey [56], a prey niche likely not available to the smaller *C. v. viridis*. Therefore, what prey species are available for consumption by sympatric rattlesnakes and the level of resource niche partitioning is determined by a number of factors, including environmentally constrained species distributions, habitat specialization, and snake size and gape limitations.

A closely related species, *C. o. oreganus*, which does not overlap *C. v. viridis* except potentially in narrow zones of Idaho, still shows a comparable wide latitudinal range, reaching from southern California to southern Canada [40]. Though this species does not extend as far south as *C. v. viridis*, a similar latitudinal shift in diet composition is observed, where mammals constitute virtually the entire diet at northern extremes and lizards represent a more significant (but still not dominant) proportion of prey consumed in the south. A study at the northern extreme (British Columbia) showed that diet consisted only of mammals (95.6%) and birds (4.4%; [57], and in British Columbia, not a single lizard was recovered from gut contents of roadkill ([58]). In Idaho, diet consisted of 98.1% mammalian, 0.9% avian, and 0.9% reptilian (represented by a single lizard; [59]) prey. Farther south in California, the diet still consisted primarily of mammals (76.1%); however, lizards comprised a larger proportion of the diet (14.8%), though juveniles consumed lizards more often than adults [60].

Venomous snakes rely on a chemical rather than a physical means of prey capture; thus, venoms that are effective at rapidly incapacitating available prey might be assumed to be highly advantageous. Indeed, broad relationships between higher venom toxicity and prey specialization in snakes have been observed in fish-eaters [61], bird-eaters [25, 37], lizard-eaters [26], and arthropod specialists [32, 62]. The evolution of these taxon-specific venoms can even lead to cases where venom is extremely toxic towards one or a few prey items and is less toxic towards others [25–27, 35, 37] due to the production of prey-specific toxins. *Crotalus v. viridis* follows this trend but in a unique way, by producing a venom dominated by myotoxin a, perhaps the only demonstrated mammal-specific toxin in a rattlesnake, in northern latitudes where mammals represent the vast majority of available prey. Purified myotoxin a is potently toxic towards mice and is known to induce rapid tetanic paralysis [49]; however, it is virtually nontoxic towards lizards, and the myotoxin-rich venoms are considerably less toxic toward lizards. Conversely, in southern areas, where nonmammals make up a larger proportion of the diet, *C. v. viridis* produces a venom phenotype effective against both lizards and mammals. This suggests (1) dietary specialization on mammals is a driving factor determining the venom phenotype of *C. v. viridis* at higher latitudes and (2) a more diverse diet with a higher proportion of non-mammalian prey in lower latitudes is related to expression of SVMP-rich venoms.

In this study, we utilize lethal toxicity as a proxy for diet-specific venom specialization. However, time-to-incapacitation as opposed to 24-h survivability may be a better overall indicator of venom functionality. While we did not quantify time-to-incapacitation during our LD_50_ experiments, we noticed rapid incapacitation due to apparent tetany of mice injected with myotoxin-rich venom or purified myotoxin a (as previously observed [49]), but we did not notice a similar effect in lizards even at high doses of purified myotoxin a. This suggests that the myotoxin-rich venom is both more immobilizing and more toxic to mice than SVMP-rich venoms. However, other factors may compensate for a minimally effective venom towards less susceptible (or more dangerous) prey types. Snakes can modify their predatory repertoire by altering post-contact release behavior, strike frequency, and distance depending on the prey type [35, 63] or by injecting a larger volume of venom [64]. Therefore, snakes in northern regions may behaviorally compensate for the lower effectiveness of their venom towards lizards, retaining their potential as a feasible prey type when encountered.

A complete view of community ecology incorporates both biotic and abiotic factors, but the abiotic processes controlling species interactions have historically been underappreciated [65]. High myotoxin and high SVMP venoms are predicted in distinct ecological niches that mirror the latitudinal shifts in phenotype we observed, indicating a relationship between environment and venom composition. We find a variety of temperature variables contributed to our ENM models (though the most significant variables differed between venom phenotype models) and that temperature fluctuations appear particularly important [20]. The overall trend suggesting that temperature is relevant to the distribution of venom phenotypes across a broad range may be explained by two non-exclusive factors: (1) the temperature-dependence of venom toxin functionality and/or (2) the importance of abiotic factors determining aspects of both predator and prey ecology and distribution [19].

SVMP-rich type 1 venoms have been suggested to play a part in prey predigestion in cooler environmental conditions [18, 66, 67]; however, their role as a digestive agent remains unclear [68, 69]. Our findings do not support a predigestive role of SVMPs in cooler environments, as SVMP-dominated degradative venoms in *C. v. viridis* are more common in warmer lower latitudes. Enzyme-dependent predigestion is optimal at higher temperatures [70] and SVMP-rich venoms are likely more efficient at southern latitudes in general. However, the repeated recovery of variables associated with temperature fluctuations (e.g., mean diurnal range [19], mean temperature of coldest quarter) in enzyme-rich Type 1 venom niche models could indicate the adaptive utility of an enzyme-rich venom in areas of greater temperature variation.

Rattlesnake predatory behavior includes strike-induced chemosensory searching [71–74], a potentially lengthy process where prey must be relocated after the classic viperid strike-and-release. This may involve following a chemical trail for hours without success, incurring the metabolic costs of activity and later venom production, which may be high regardless of external conditions [75, 76]. Furthermore, it is possible that in cooler northern climates, snakes have a slower response time during predatory events [77], which may also be problematic for prey relocation. In *C. cerastes*, less than half of bites result in successful subjugation and feeding in part due to the antipredator maneuvers displayed by some rodent prey that cause premature fang withdrawal and incomplete envenomation [78, 79]. Venom that rapidly incapacitates mammals (virtually the only food source at northern extremes) even at low doses via the mammal-specific myotoxin a would provide an adaptive edge over SVMP-rich venoms since they require a lower volume of venom and less chemosensory searching, resulting in more efficient feeding for low-metabolism snakes in cooler climates.

A more likely scenario is that temperature-related abiotic variables that shift with latitude have a cascading influence on prey distribution and therefore have an indirect impact on numerous toxin families that define venom phenotypes. Latitude is a major defining factor in the distribution of nearly all species [50]; however, global ectotherm species richness is more strongly related to latitudinal shifts in temperature [52, 53]. Due to the steep decline in ectotherm diversity as climactic temperatures decrease, snakes become increasingly reliant on mammalian prey at higher latitudes. Our data suggests that this pressure drove the evolution of mammal-specific toxicity in northern venoms and generalist toxicity in southern venoms. This points to dietary availability, as determined by latitudinal temperature gradients, as a key driving factor shaping the geographic variation in venom composition. The fact that model performance predicting venom composition increased with the addition of environmental variables to species richness suggests the importance of abiotic factors combined with dietary availability in shaping venom composition. The majority of environmental variables that predicted venom phenotype also predicted species richness, suggesting that venom phenotype is largely indirectly impacted by environment and is due to the more direct influence of abiotic variables on prey availability.

## Conclusions

Decades of research has contributed to our contemporary understanding that snake venom composition is remarkably variable between and even within species [8–22], yet the biotic and abiotic factors that drive this extreme variation have remained largely speculative [13, 18–20, 80]. Our findings provide another key example detailing the extensive venom variation within a single species. We find evidence that venom variation correlates both with abiotic climatic factors and prey type distributions and that variation in venom composition may be linked to differential toxicity for distinct prey taxa (i.e., reptiles versus mammals). These links between venom variation and variation in biotic and abiotic factors further suggest that venom variation likely results from substantial geographic variation in selection regimes that dictate the efficacy of venom phenotypes at the toxin family level across the range of snake species. This highlights the implicit influence of abiotic factors on venom phenotype and provides further evidence for a dominant role of local selection as a key driver of venom variation.

## Methods

### Snake collection and venom extraction

Snakes and venom samples were field collected over a five-year period across the central United States from nine states that span the wide latitudinal range of *C. v. viridis* (Fig. 1). Snakes were either field-extracted or processed in the UNC Animal Resource Facility and returned to their location of capture within 3 days, or they were maintained in the UNC Animal Resource Facility per UNC IACUC approval (protocol #1302D-SM-S-16). All snake collections and field-collected samples were conducted according to permits obtained from Colorado Parks and Wildlife (scientific collecting permits #14HP974, 17HP0974, 20HP0974, to SPM), Wyoming Game and Fish Department (scientific collecting permit #1544, SPM), New Mexico Game and Fish Department (permit #3418, SPM), Utah Division of Wildlife Resources (permit #1COLL10567, SPM), Texas Parks and Wildlife Department (permit #SPR-0604–391, SPM), and Montana Fish, Wildlife and Parks (permits 2019–075-W and 2020–076-W, TAC). Venom was manually extracted as described [81]; samples were either dried in the field over calcium carbonate and placed on ice until storage at − 20 °C or were placed in liquid nitrogen until lyophilization and storage at − 20 °C. Due to the potential for ontogenetic venom variation, only venoms from adult snakes were utilized in this study.

### Protein concentration determination

Dried venom samples were reconstituted at an approximate concentration of 4.0 mg/mL in Millipore-filtered water, vortexed, and centrifuged for 5 min at 9500 × g. Samples were diluted to an approximate concentration of 0.5 mg/mL and centrifuged again for 5 min at 9500 × g. Protein concentration was then determined in triplicate with a Nanodrop using the Absorbance 280 protocol, and 260/280 ratio was recorded. The amount of material used in all subsequent assays was based on these determinations. Reconstituted stock samples at 4 mg/mL were frozen at − 20 °C until use and then thawed and centrifuged at 9500 × g for 5 min to pellet cellular debris.

### Enzymology

#### Azocasein metalloprotease assay

The azocasein metalloprotease assay protocol was performed as outlined in Aird and da Silva [82]. Briefly, 20 μg of venom was incubated with 1.0 mg of azocasein substrate in 0.5 mL buffer (50 mM HEPES, 100 mM NaCl, pH 8.0) for 30 min at 37 °C. The reaction was stopped with 250 μl of 0.5 M trichloroacetic acid, vortexed, brought to room temperature, and centrifuged at 2000 rpm (~ 700 × g) for 10 min. Absorbance of the supernatant was read at 342 nm, and all values were expressed as ΔA_342nm_/min/mg venom protein.

#### L-amino acid oxidase assay

L-amino acid oxidase assays were performed as outlined in Weissbach [83]. L-kynurenine substrate was solubilized at 1.04 mg/mL buffer (50 mM HEPES, 100 mM NaCl, pH 8.0), and 75 μl was added to 20 μg of venom in 645 μl buffer. The reaction was incubated at 37 °C for 30 min and then terminated with 750 μl of 10% trichloroacetic acid. Absorbances were read at 331 nm and specific activity was calculated as nanomoles product formed/min/mg venom protein from a standard curve of the reaction product, kynurenic acid, using the same buffer and acid termination conditions as indicated above.

#### Phosphodiesterase assay

Phosphodiesterase assays were performed based on Laskowski’s [84] modification of Björk [85]. Twenty micrograms of crude venom (5 μL) was added to 220 μl of buffer (100 mM tris–HCl, 10 mM MgCl_2_, pH 9.0). One hundred fifty μl of 1.0 mM bis-p-nitrophenylphosphate substrate was added, and the reaction was incubated at 37 °C for 30 min. The reaction was terminated with 375 μl of 100 mM NaOH containing 20 mM disodium-EDTA, vortexed, brought to room temperature and absorbance read at 400 nm. Specific activity was expressed as ΔA_400nm_/min/mg venom protein.

#### Thrombin-like and kallikrein-like serine protease assays

Serine protease assays were performed as described by Mackessy [86]. Eight μg of crude venom (2 μL) was added to 373 μl of buffer (50 mM HEPES, 100 mM NaCl, pH 8.0). Tubes were incubated at 37 °C for approximately 3 min. Fifty μl of thrombin-like substrate (1.0 mM BzPheValArg-pNA; Sigma) or kallikrein-like substrate (1.0 mM Bz-ProPheArg-pNA; Bachem) was added and the sample was vortexed and placed back at 37 °C. Reactions were stopped after 5.0 min with 50% acetic acid. The absorbances for each of the samples were read at 405 nm, and specific activity was calculated from a standard curve of p-nitroaniline and expressed as nanomoles product produced/min/mg of venom.

#### Phospholipase A_2_ assay

Phospholipase A_2_ assays were performed as outlined in Holzer and Mackessy [87]. Briefly, 12.5 μL of crude venom was combined with 583 μL of assay buffer (10 mM tris–HCl, pH 8.0 with 10 mM CaCl_2_, 100 mM NaCl) followed by 50 μL of 4-nitro-3-(octanoyloxy)benzoic acid substrate. Samples were incubated at 37 °C in duplicate for 20 min and the reaction was stopped with 50 μL of 2.5% Triton X-100. Samples were read at 425 nm and specific activity was recorded as nmol product/min/mg venom protein.

#### High-performance liquid chromatography

One milligram of venom was subjected to reverse phase HPLC using a Waters system, Empower software, and a Phenomenex Jupiter C_18_ (250 × 4.6 mm, 5 μm, 300 Å pore size) column as outlined in Smith and Mackessy [88]. Protein/peptide was detected at 280 nm and 220 nm with a Waters 2487 Dual λ Absorbance Detector. Fractions corresponding to each peak were then frozen at − 80 °C overnight, lyophilized and then analyzed along with 20 μg crude venom via SDS-PAGE as described [88] in order to determine peak complexity, mass and toxin families [43]. Percent peak area at 280 nm was recorded as a proxy for relative toxin abundance.

#### Myotoxin a purification

Based on protein size (SDS-PAGE), peak 5 was identified as myotoxin-containing and was further purified using reverse phase high-performance liquid chromatography using a Phenomenex Jupiter C18 (250 × 4.6 mm, 5 mm, 300 Å pore size) column, and 1-min fractions were collected at a flow rate of 1 mL/min for a total of 61 min. To elute proteins, a gradient of 95% solution A (0.1% trifluoroacetic acid in Millipore-filtered water) to 100% solution B (0.1% TFA in 100% acetonitrile) was used (5% solution B for 6 min; 5–22% B over 2 min; 22–35% B over 37 min; 35–100% over 2 min; and returning to 5% B over 2 min). Protein/peptide was detected at 220 nm and 280 nm with a Waters 2487 Dual l Absorbance Detector. Fractions corresponding to each peak were pooled, then frozen overnight at − 80 °C and lyophilized.

The dominant peak at 21 min and a secondary peak at 28 min were analyzed via Bruker Microflex LRF MALDI-TOF Proteomics (Proteomics and Metabolomics Facility at Colorado State University (Fort Collins, CO)) to confirm isoform mass identity previously determined by Griffin and Aird [89]. Approximately 1.0 mg protein was dissolved in 1.0 mL 50% acetonitrile containing 0.1% TFA, mixed with 1.0 mL sinapinic acid matrix (10 mg/mL, dissolved in the same solvent), and spotted onto target plates.

### Statistical analyses

A Mantel test was run using Euclidean distance measures in PC-ORD 7.07 [90] for all toxins (using specific enzyme activity or toxin abundance based on HPLC) in order to assess the association between venom components and geographic location (*p* < 0.05). The PC-ORD enzyme specific activities were normalized to the maximum value for each toxic activity. Linear regression was performed in GraphPad Prism 9® between all enzyme activities and toxin abundances with latitude to determine if toxins varied significantly over latitude. Linear regression *t*-tests were run to determine if the regression line slope differed significantly from 0. Cluster analysis was run in PC-ORD 7.07 [90] with a Euclidean Distance Measure and Ward’s Group Linkage Method to bin samples into defined venom phenotypes based on the venom toxin abundances and activities that were significant (*p* < 0.05) both the Mantel test and the linear regressions.

We investigated the diet composition in the geographic ranges that corresponded to the identified venom phenotype groups and performed discriminant analysis in SPSS® to test group membership using latitude as a predictor and bootstrapping with a 95% confidence interval. In order to test the relationship between diet groups and venom variables that significantly differed geographically, we performed a one-way ANOVA in SPSS® with a significance level of 0.05.

#### Shotgun proteomics

Eight representative venom samples from New Mexico, Colorado, and Montana were subjected to shotgun proteomics. Venoms (20 μg) were resuspended in 8 M urea/0.1 M Tris (pH 8.5) and reduced with 5 mM TCEP (tris (2-carboxyethyl) phosphine) for 20 min at room temperature. Samples were alkylated with 50 mM 2-chloroacetamide for 15 min in the dark at room temperature and then diluted fourfold with 100 mM Tris–HCl (pH 8.5) and trypsin digested at an enzyme/substrate mass ratio of 1:20 overnight at 37 °C. To stop the reaction, samples were acidified with formic acid (FA), and digested peptides were purified with Pierce™ C18 Spin Tips (Thermo Scientific) according to the manufacturer’s protocol. Samples were dried in a speed vacuum and resuspended in 0.1% FA. Liquid chromatography tandem mass spectrometry (LC–MS/MS) was performed using an Easy nLC 1000 instrument coupled with a Q Exactive™ HF Mass Spectrometer (both from ThermoFisher Scientific). For each venom, 3 μg of digested peptides were loaded on a C18 column (100 μM inner diameter × 20 cm) packed in-house with 2.7 μm Cortecs C18 resin and separated at a flow rate of 0.4 μl/min with solution A (0.1% FA) and solution B (0.1% FA in ACN) under the following conditions: isocratic at 4% B for 3 min, followed by 4–32% B for 102 min, 32–55% B for 5 min, 55–95% B for 1 min and isocratic at 95% B for 9 min. Mass spectrometry was performed in data-dependent acquisition (DDA) mode. Full MS scans were obtained from *m/z* 300 to 1800 at a resolution of 60,000, an automatic gain control (AGC) target of 1 × 10^6^, and a maximum injection time (IT) of 50 ms. The top 15 most abundant precursors with an intensity threshold of 9.1 × 10^3^ were selected for MS/MS acquisition at a 15,000 resolution, 1 × 10^5^ AGC, and a maximal IT of 110 ms. The isolation window was set to 2.0 m*/z* and ions were fragmented at a normalized collision energy of 30. Dynamic exclusion was set to 20 s.

Fragmentation spectra were searched against an in-house *Crotalus* database containing translated sequences derived from multiple venom transcriptomes (derived from public databases) using the MSFragger-based FragPipe computational platform [91, 92]. Reverse decoys and contaminants were included in the search database. Cysteine carbamidomethylation was selected as a fixed modification, oxidation of methionine was selected as a variable modification, and precursor-ion mass tolerance and fragment-ion mass tolerance were set at 20 ppm and 0.4 Da, respectively. Up to 2 missed tryptic cleavages were allowed and the protein-level false discovery rate (FDR) was set to < 1%. The relative abundance of major toxin families was compared across samples using log-transformed total spectral intensity [93]. Raw mass spectrometry proteomics data has been deposited to the Mass Spectrometry Interactive Virtual Environment (MassIVE) repository with the dataset identifier MSV000089601.

### Phylogeographic and population genetic analyses

We incorporated previously published and analyzed ddRADseq data [94] to investigate how phylogeographic and population genetic structure correspond with patterns of venom variation (Additional file 5: Table S5; see 94 for details). We mapped individual samples to the *C. viridis* reference genome [95] using the program BWA v.0.7.10 [96] specifying the “mem” option with default settings. We then used a combination of SAMtools and BCFtools to generate pileup alignments of all samples and called variant sites [97]. We filtered out sites that had read depths lower than 5 and retained only biallelic SNPs that were present across at least 90% of samples using VCFtools. We used the program BEAST V.2.5.3 [98] to infer phylogenetic relationships from our SNP dataset by using BEAUti2 to format input files using a JC69 substitution model and a Gamma Site model. We performed 10,000,000 MCMC iterations with a burn-in of 100,000 generations under a Yule model prior. We included data from several samples of *C. v. nuntius*, historically considered a subspecies of *C. viridis*, and we designated *C. oreganus concolor* (a closely related sister species) as the outgroup and visualized phylogenetic trees using TreeAnnotator to produce a maximum clade credibility tree with posterior probabilities (PP) to assess node support. To evaluate population genetic structure, we used the program STRUCTURE [99] to infer probabilities of individuals of being assigned to *K* genetic clusters. STRUCTURE uses a Bayesian clustering approach on multilocus genotype data both to identify populations and to assign individuals to a population [99]. For multiple values of *K* (two through four), we ran 20 iterations using the program StrAuto [100] with 10,000 burn-in generations followed by 75,000 logged generations. We first used the Evanno method which examines the overall change in likelihood scores across *K* values to determine the most supported value (Δ*K*; [101]) and then combined iterations from the highest supported *K* value using the “greedy” method in the program CLUMPP [102] for each *K* value.

### Dietary analysis

Natural history collections in multiple locations containing fluid-preserved Prairie Rattlesnake specimens were visited to examine specimens (Additional file 6: Table S6). Specimens were considered usable if accompanying data did not indicate that animals were captive or were held in captivity prior to preservation. Before sampling for prey remains, snakes were sexed via presence or absence of hemipenes, and snout-vent length (SVL) and tail length (TL) were recorded by measuring snakes with a soft metric tape measure.

Prey remains were sampled via inspection of stomach contents by making a small (2–6 cm) midventral incision through ventral scales of the snake. Once located, an additional incision was made through the stomach wall to determine if a food bolus was present. If present, direction of ingestion of the prey item was recorded (inferred from orientation in the stomach), and the bolus was removed for identification to greatest taxonomic resolution possible. For the purposes of this study, prey remains were identified based on the presence of fur, scales, or feathers into broad categories (amphibian, reptile, mammal, bird) and were stored in 70% ethanol.

### Lethal toxicity (LD_50_) assays

Venoms from snakes found in Lincoln County, Colorado (to represent the southern venom phenotype), Weld County, Colorado (to represent the northern venom phenotype), and purified myotoxin a were tested against NSA mice and *Anolis* sp. lizards. Doses from 0 to 10 μg/g (3 mice/dose) were given with an intraperitoneal injection (100 μl bolus of 0.9% saline for mice, 50 μl for lizards) on the right side of the body. NSA mice were used because we have bred them in our facility for many years and they are traceable to Jackson Labs, they originate from an outcrossed line, and they have been a standard strain for much of our research over the last 20 + years; hence, toxicity data of venoms from different species are comparable (same model strain). Mouse LD_50_ determinations used the Trimmed Spearman–Karber (TSK) program version 1.5 (U.S. Environmental Protection Agency, 1990). Toxicity data was analyzed using “Quest Graph™ LD50 Calculator,” AAT Bioquest, Inc., 1 Feb. 2023. [https://www.aatbio.com/tools/ld50-calculator] For lizards, additional myotoxin a doses of 25 and 50 μg/g were administered in a 50 μL bolus.

### Environmental niche modeling

To determine if differences in venom composition correlated with environmental variables, ecological niche models (ENMs) were constructed in MAXENT v3.4.1 [103] for the occurrence of venom phenotypes (as determined by cluster analysis of toxins with significant geographic variation). MAXENT uses occurrence points and a set of input environmental variables to generate a probability of presence distribution map [103]. Locality points were filtered in the R package *spThin* to a minimum nearest neighbor distance of 10 km in order to reduce sampling bias and improve model performance [104]. Environmental variables were taken from the WorldClim dataset v 1.4 [105] with a 1-km resolution. WorldClim data was processed in ArcGIS Pro to the region of interest, and all layers were modified to the same cell size (1 km), extent, and projected coordinate system. Highly correlated environmental variables (BIO 3, BIO 5, BIO 6, BIO 7, BIO 13, BIO 14, BIO 16), including those that were calculated based on other variables, were removed to avoid biasing model fitting [19, 106]. MAXENT was run with 15 replicates and 5000 iterations. Cross-validation with random seeding was used to determine the best model and area under the curve (AUC) was used to evaluate model performance.

In order to investigate further the relationship between environment and diet with venom composition, we extracted WorldClim dataset v1.4 environmental variables and species richness values from raster layers that corresponded to exact collection localities of our 147 adult samples used for venom analysis [105, 107] and linked these to each venom sampling locality in ArcGIS Pro 3.0. We tested the relationship between diet and environment on all venom components individually with hierarchical linear regressions between each venom component and predictor variables from two models: (1) mammal and reptile species richness alone [107] and (2) mammal and reptile species richness combined with the WorldClim dataset v 1.4 described above [105]. We also performed linear regression to test the relationship between WorldClim variables and mammal and reptile species richness and further investigated the relationship between dietary availability and venom phenotype with a multinomial logistic regression with a chi-square goodness-of-fit test and a confidence interval of 95% between mammal and reptile species richness [107] with both venom phenotypes.

## Supplementary Information

**Additional file 1: Table S1.** Venom Sample Information. Collection information for all individuals included in study.

**Additional file 2:** **Table S2.** Proteomics Data. List of venom proteins identified by LC-MS/MS with total signal intensity values for each protein. **Table S3.** Proteomics Data. List of venom toxin families identified by LC-MS/MS with the summed total signal intensity values for all proteins in a family.

**Additional file 3:** **Figure S1.** Enzyme Activities. Linear regressions of latitude with major enzyme toxins. a) Azocasein snake venom metalloprotease*, b) Thrombin-like serine protease*, c) Kallikrein-like serine protease*, d) Phosphodiesterase*, e) Phospholipase A2 and f) L-amino acid oxidase. * = *p*<0.05.

**Additional file 4:** **Table S4.** Environmental Modeling. Percent contributions of WorldClim environmental layers to ENM predictions.

**Additional file 5:** **Table S5.** Genomic Information. Species ID, project ID, locality, tree label, SRA accession, and reference study for genomic data.

**Additional file 6:** **Table S6.** Diet Data Collection Information. Museum collections investigated, collection location, and number of specimens examined.

## Data Availability

All data generated or analyzed during this study are included in this published article, its supplementary information files and publicly available repositories. Proteomics results are available in Additional file 2: Tables S2-S3. Raw mass spectrometry proteomics data has been deposited to the Mass Spectrometry Interactive Virtual Environment (MassIVE) repository with the dataset identifier MSV000089601. The address is as follows: https://massive.ucsd.edu/ProteoSAFe/datasets.jsp#%7B%22query%22%3A%7B%7D%2C%22table_sort_history%22%3A%22createdMillis_dsc%22%7D and the ID number is MSV000089601. Information is also available through Proteome Xchange with the dataset identifier PXD034274 (http://proteomecentral.proteomexchange.org/cgi/GetDataset?ID=PXD034274). Final alignment files for genetic analyses can be found on Dryad (10.5061/dryad.3j9kd51ph).

## Abbreviations

SVMP: Snake venom metalloprotease
SVSPs: Snake venom serine protease
PLA_2_: Phospholipase A_2_
L-AAO: L-amino acid oxidase
PDE: Phosphodiesterase
ENM: Environmental niche modeling
HPLC: High-performance liquid chromatography
SVL: Snout-vent length
TL: Tail length
AUC: Area under the curve
CRiSP: Cysteine-rich secretory protein
CTL: C-type lectin
