# Supplementary material for: Snakes on a plain: biotic and abiotic factors determine venom compositional variation in a wide-ranging generalist rattlesnake
Source: BMC Biol. 2023 Jun 6;21:136. doi: 10.1186/s12915-023-01626-x (PMC10246093; doi:10.1186/s12915-023-01626-x)
Supplement: Supplementary file 3 — Additional file 3: Figure S1. Enzyme Activities. Linear regressions of latitude with major enzyme toxins. a) Azocasein snake venom metalloprotease*, b) Thrombin-like serine protease*, c) Kallikrein-like serine protease*, d) Phosphodiesterase*, e) Phospholipase A2 and f) L-amino acid oxidase. * = p<0.05. [file 12915_2023_1626_MOESM3_ESM.docx]

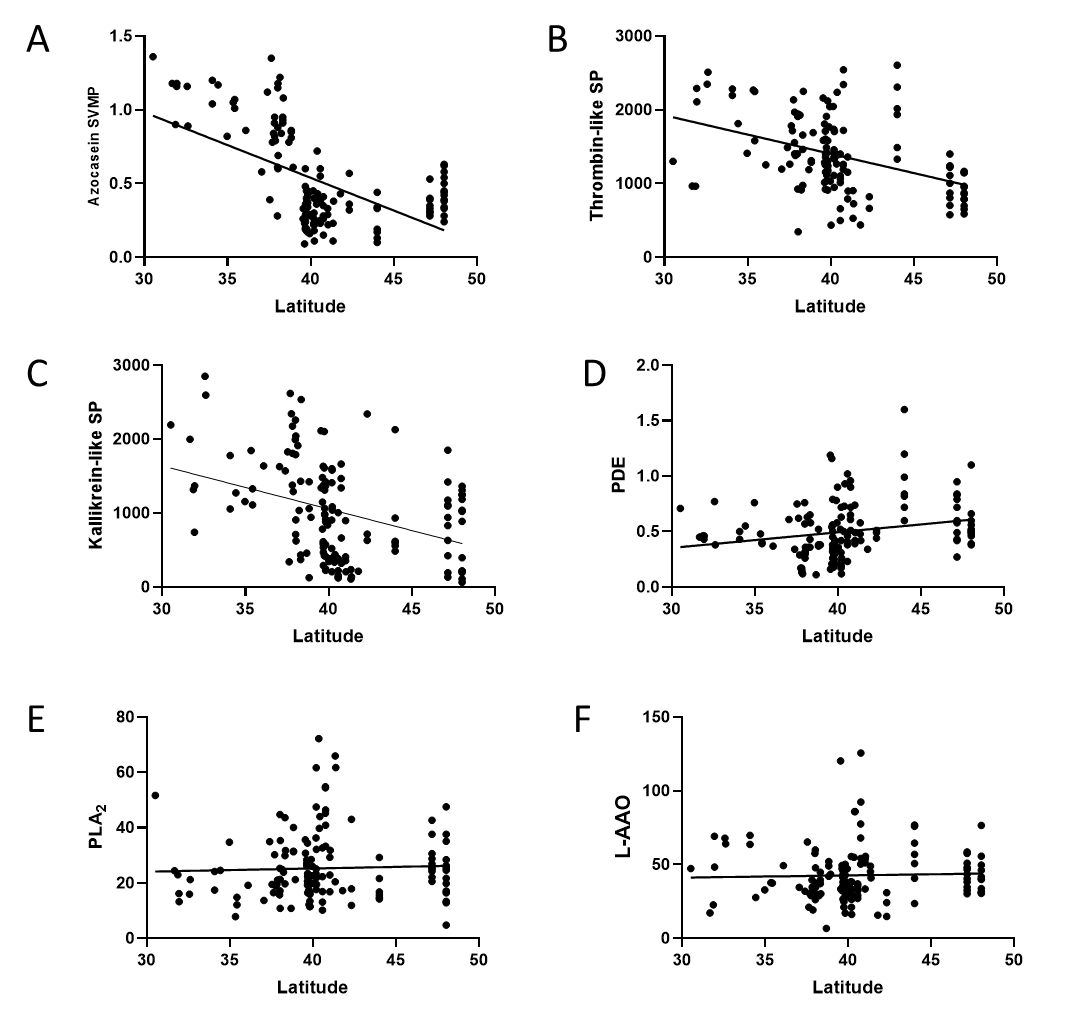


Supplemental Figure S1. Linear regressions of latitude with major enzyme toxins (specific activities).

a) Azocasein snake venom metalloprotease*, b) Thrombin-like serine protease*, c) Kallikrein-like serine protease*, d) Phosphodiesterase*, e) Phospholipase A_2_ and f) L-amino acid oxidase. * = p<0.05.
