# Supplementary material for: Snakes on a plain: biotic and abiotic factors determine venom compositional variation in a wide-ranging generalist rattlesnake
Source: BMC Biol. 2023 Jun 6;21:136. doi: 10.1186/s12915-023-01626-x (PMC10246093; doi:10.1186/s12915-023-01626-x)
Supplement: Supplementary file 4 — Additional file 4: Table S4. Environmental Modeling. Percent contributions of WorldClim environmental layers to ENM predictions. [file 12915_2023_1626_MOESM4_ESM.docx]

Supplemental Table S4. Percent contributions of WorldClim environmental layers to ENM predictions.

| Layer | Variable | Percent contribution to northern range | Layer | Variable | Percent contribution to southern range |
| --- | --- | --- | --- | --- | --- |
| bio_9 | mean temp driest quarter | 24.4 | bio_2 | mean diurnal range | 43.9 |
| bio_1 | annual mean temperature | 17.8 | bio_11 | mean temperature of coldest quarter | 21.8 |
| bio_2 | mean diurnal range | 15.6 | bio_19 | precipitation of coldest quarter | 8.8 |
| bio_4 | temperature seasonality | 9.5 | bio_12 | annual precipitation | 8.2 |
| bio_11 | mean temperature of coldest quarter | 9.4 | bio_4 | temperature seasonality | 7 |
| bio_18 | precipitation of warmest quarter | 9.3 | bio_18 | precipitation of warmest quarter | 2.6 |
| bio_15 | precipitation seasonality | 5.8 | bio_8 | mean temperature of wettest quarter | 2.1 |
| bio_8 | mean temperature of wettest quarter | 3.1 | bio_10 | mean temperature of warmest quarter | 1.9 |
| bio_12 | annual precipitation | 3 | bio_15 | precipitation seasonality | 1.8 |
| bio_19 | precipitation of coldest quarter | 1.6 | bio_17 | precipitation of driest quarter | 0.9 |
| bio_10 | mean temperature of warmest quarter | 0.5 | bio_9 | mean temperature of driest quarter | 0.9 |
| bio_17 | precipitation of driest quarter | 0 | bio_1 | annual mean temperature | 0.1 |
