# Supplementary material for: Snakes on a plain: biotic and abiotic factors determine venom compositional variation in a wide-ranging generalist rattlesnake
Source: BMC Biol. 2023 Jun 6;21:136. doi: 10.1186/s12915-023-01626-x (PMC10246093; doi:10.1186/s12915-023-01626-x)
Supplement: Supplementary file 5 — Additional file 5: Table S5. Genomic Information. Species ID, project ID, locality, tree label, SRA accession, and reference study for genomic data. [file 12915_2023_1626_MOESM5_ESM.docx]

| **Table S5**. **Species ID, project ID, locality, tree label, SRA accession, and reference study** | | | | | | |
| --- | --- | --- | --- | --- | --- | --- |
| **Taxon** | **Project ID** | **State** | **County** | **Tree Label (see Fig. 4)** | **SRA Accession** | **Reference** |
| *C. v. viridis* | CV0004 | Colorado | Weld | Northern Colorado | SAMN12003329 | Schield & Perry et al. 2019 |
| *C. v. viridis* | CV0005 | Colorado | Weld | Northern Colorado | SAMN12003330 | Schield & Perry et al. 2019 |
| *C. v. viridis* | CV0006 | Colorado | Weld | Northern Colorado | SAMN12003331 | Schield & Perry et al. 2019 |
| *C. v. viridis* | CV0007 | Colorado | Weld | Northern Colorado | SAMN09394451 | Schield et al. 2018 |
| *C. v. viridis* | CV0008 | Colorado | Weld | Northern Colorado | SAMN12003332 | Schield & Perry et al. 2019 |
| *C. v. viridis* | CV0009 | Colorado | Weld | Northern Colorado | SAMN12003333 | Schield & Perry et al. 2019 |
| *C. v. viridis* | CV0010 | Colorado | Weld | Northern Colorado | SAMN12003334 | Schield & Perry et al. 2019 |
| *C. v. viridis* | CV0011 | Colorado | Weld | Northern Colorado | SAMN12003335 | Schield & Perry et al. 2019 |
| *C. v. viridis* | CV0012 | Colorado | Weld | Northern Colorado | SAMN12003336 | Schield & Perry et al. 2019 |
| *C. v. viridis* | CV0013 | Colorado | Weld | Northern Colorado | SAMN12003337 | Schield & Perry et al. 2019 |
| *C. v. viridis* | CV0017 | New Mexico | Dona Ana | Southern New Mexico | SAMN09394452 | Schield et al. 2018 |
| *C. v. viridis* | CV0018 | New Mexico | Luna | Southern New Mexico | SAMN12003339 | Schield & Perry et al. 2019 |
| *C. v. nuntius* | CV0075 | Arizona | Coconino | *C . v. nuntius* | SAMN12003366 | Schield & Perry et al. 2019 |
| *C. v. nuntius* | CV0077 | Arizona | Navajo | *C . v. nuntius* | SAMN12003368 | Schield & Perry et al. 2019 |
| *C. v. viridis* | CV0173 | Colorado | Moffat | Northern Colorado | SAMN12003412 | Schield & Perry et al. 2019 |
| *C. v. viridis* | CV0183 | Colorado | Costilla | Southern Colorado | SAMN12003422 | Schield & Perry et al. 2019 |
| *C. v. viridis* | CV0190 | Colorado | Baca | Southern Colorado | SAMN12003429 | Schield & Perry et al. 2019 |
| *C. v. nuntius* | CV0191 | New Mexico | McKinley | *C . v. nuntius* | SAMN12003430 | Schield & Perry et al. 2019 |
| *C. v. viridis* | CV0197 | Colorado | Park | Northern Colorado | SAMN12003436 | Schield & Perry et al. 2019 |
| *C. v. viridis* | CV0215 | Colorado | Weld | Northern Colorado | SAMN12003442 | Schield & Perry et al. 2019 |
| *C. v. viridis* | CV0216 | Colorado | Larimer | Northern Colorado | SAMN12003443 | Schield & Perry et al. 2019 |
| *C. v. viridis* | CV0219 | Montana | Otero | Southern Colorado | SAMN12003445 | Schield & Perry et al. 2019 |
| *C. v. viridis* | CV0221 | Montana | Chouteau | Montana | SAMN12003446 | Schield & Perry et al. 2019 |
| *C. v. viridis* | CV0222 | Montana | Chouteau | Montana | SAMN12003447 | Schield & Perry et al. 2019 |
| *C. v. viridis* | CV0230 | Montana | Chouteau | Montana | SAMN12003450 | Schield & Perry et al. 2019 |
| *C. v. viridis* | CV0231 | Montana | Chouteau | Montana | SAMN12003451 | Schield & Perry et al. 2019 |
| *C. v. viridis* | CV0232 | Montana | Chouteau | Montana | SAMN12003452 | Schield & Perry et al. 2019 |
| *C. v. viridis* | CV0317 | Nebraska | Dawes | Nebraska | SAMN09394453 | Schield et al. 2018 |
| *C. v. viridis* | CV0320 | Oklahoma | Woods | Oklahoma | SAMN09394454 | Schield et al. 2018 |
| *C. v. viridis* | CV0321 | Texas | Culberson | Texas | SAMN12003459 | Schield & Perry et al. 2019 |
| *C. v. viridis* | CV0322 | Texas | Glasscock | Texas | SAMN12003460 | Schield & Perry et al. 2019 |
| *C. v. viridis* | CV0568 | New Mexico | Torrence | Northern New Mexico | SAMN12003508 | Schield & Perry et al. 2019 |
| *C. v. viridis* | CV0570 | New Mexico | Torrence | Northern New Mexico | SAMN12003510 | Schield & Perry et al. 2019 |
| *C. v. viridis* | CV0571 | New Mexico | Torrence | Northern New Mexico | SAMN12003511 | Schield & Perry et al. 2019 |
| *C. v. viridis* | CV0572 | New Mexico | Torrence | Northern New Mexico | SAMN12003512 | Schield & Perry et al. 2019 |
