# Supplementary material for: Snakes on a plain: biotic and abiotic factors determine venom compositional variation in a wide-ranging generalist rattlesnake
Source: BMC Biol. 2023 Jun 6;21:136. doi: 10.1186/s12915-023-01626-x (PMC10246093; doi:10.1186/s12915-023-01626-x)
Supplement: Supplementary file 6 — Additional file 6: Table S6. Diet Data Collection Information. Museum collections investigated, collection location, and number of specimens examined. [file 12915_2023_1626_MOESM6_ESM.docx]

Supplemental Table S6. Museum collections investigated, collection location, and number of specimens examined.

| Collection Name (Abbreviation) | Location | # Specimens examined |
| --- | --- | --- |
| University of Northern Colorado (UNCO) | Greeley, Colorado | 189 |
| Sternberg Museum of Natural History (FHSM) | Hays, Kansas | 178 |
| Monte L. Bean Life Science Museum (BYU) | Provo, Utah | 35 |
| Sam Noble Oklahoma Museum of Natural History (OMNH) | Norman, Oklahoma | 21 |
| Amphibian and Reptile Diversity Research Center (UTA) | Arlington, Texas | 18 |
| University of Wyoming Museum of Vertebrates (UWYMV) | Laramie, Wyoming | 10 |
| Museum of Southwest Biology (UNM) | Albuquerque, New Mexico | 185 |
